# Comparison of Perceived and Observed Hand Hygiene Compliance in Healthcare Workers in MERS-CoV Endemic Regions

**DOI:** 10.3390/healthcare6040122

**Published:** 2018-10-07

**Authors:** Modhi Alshammari, Kelly A. Reynolds, Marc Verhougstraete, Mary Kay O’Rourke

**Affiliations:** Mel and Enid Zuckerman College of Public Health, Department of Community, Environment and Policy, University of Arizona, Tucson, AZ 85719, USA; reynolds@email.arizona.edu (K.A.R.); mverhougstraete@email.arizona.edu (M.V.); MKOR@email.arizona.edu (M.K.O.)

**Keywords:** hand hygiene, healthcare, viruses

## Abstract

This study investigated healthcare workers’ perceptions of hand hygiene practices by comparing personal reports, as assessed by questionnaires, to direct observations of the workers’ hand hygiene practices. The study employed a cross-sectional research design. Observations were made using a 16-item checklist, based on three sources: Centers for Disease Control and Prevention (CDC), World Health Organization (WHO), and Boyce and Pittet’s guidelines of hand hygiene. The checklist was used for both direct-observation and self-reported data collection purposes. Pearson correlation and Multivariate Analysis of Covariance (MANCOVA) were utilized to statistically determine the relationship between healthcare workers’ reports of hand hygiene practices and observed hand hygiene behaviors. The study was conducted in the outpatient examination rooms and emergency departments of three types of hospitals in the Eastern region of Saudi Arabia where Middle East respiratory syndrome coronavirus (MERS-CoV) is endemic and is observed in routine cases and outbreaks. The total sample size included 87 physicians and nurses recruited while on duty during the scheduled observation periods, with each healthcare worker being observed during individual medical examinations with at least three patients. No statistically significant correlations between the healthcare workers’ perceptions of hand hygiene practices and healthcare workers’ actual behaviors were evident. Based on the self-report questionnaires, significant differences were found between physicians’ and nurses’ hand hygiene practices reports. Healthcare workers clearly understand the importance of careful hand hygiene practices, but based on researchers’ observations, the medical personnel failed to properly implement protocol-driven hand hygiene applications. However, the significant differences between physicians’ and nurses’ self-reports suggest further inquiry is needed to fully explore these discrepancies.

## 1. Introduction

### 1.1. Middle East Respiratory Syndrome (MERS)

Middle East respiratory syndrome (MERS) is a type of coronavirus first discovered in Saudi Arabia in 2012 [1,2]. The virus causes a range of respiratory and gastrointestinal symptoms including fever, cough, shortness of breath, and diarrhea. Infections may progress to pneumonia and organ failure leading to approximately 35% reported mortality rate [3].

MERS in the region was previously attributed to local bats. Recent studies indicated that animal to human transmission is a cause, but with camels, instead of bats, as the most likely source [4]. Per an August 2017 Ministry of Health Report 4, the MERS coronavirus has infected a total of 1609 people with 685 fatalities in Saudi Arabia [5]. Chowell et al. conclude that a significant number of MERS cases are linked to healthcare settings, ranging from 43.5% for the outbreak in Jeddah, Saudi Arabia, in 2014, to 100% for the outbreaks in Al-Hasa, Saudi Arabia, in 2013 [6].

### 1.2. Transmission of Virus in Hospitals

MERS, a droplet-transmitted virus, is primarily transmitted by contact with surfaces or patients already infected with the virus. Patients with MERS can present with mild and atypical symptoms, making it hard to identify the virus from the initial medical visit. Healthcare workers, in order to protect themselves and other patients, should practice standard precautions, including hand-washing hygiene before and after patient contact, use of personal protective equipment, adequate sterilization of patient care equipment before subsequent use, and respiratory etiquette (giving masks to patients with coughs and encouraging patients to appropriately cover their mouths) [7].

The World Health Organization proclaimed hand hygiene to be the major component of standard precaution and one of the most effective methods to prevent transmission of pathogens. Specifically, hand hygiene practice refers to washing hands with plain soap and water, using water alone, or rubbing hands with an alcohol-based solution [8]. The MERS outbreak in Saudi Arabia raised questions about the potential exposure to the virus, as well as the hygiene protocols followed by healthcare workers. Numerous researchers concur that effective hand hygiene practices could significantly limit the transmission of MERS and other emerging viruses in endemic regions [2,7,9]. In Western medical practice, patients enter a waiting room and check in at the reception. They wait and are eventually escorted to a small exam room. Healthcare providers usually use either hand sanitizer or wash hands when entering the room with a new patient. In Saudi Arabia, a doctor is assigned a stationary examination room where he/she waits at a desk and successive patients enter the room. The physician may not move and lacks the physical prompt to implement hand hygiene practices between patients. Hand hygiene compliance among healthcare workers has been thoroughly studied by questionnaire [10,11] and direct observations [12,13]. Additionally, a few studies have examined a combination of questionnaires and observations [14,15]. The current research investigates the differences noted between healthcare workers’ self-reports of hand hygiene practices and researcher observations of actual hand hygiene behavior.

## 2. Materials and Methods

Quantitative data was collected using a dichotomous scale, with questionnaire data utilizing a five-level Likert scale [16] to measure each participant’s hand hygiene reports and behavior.

### 2.1. Setting

The study took place in three types of hospitals, i.e., public, security forces, and private, in Eastern Saudi Arabia, with approval of the study design and instruments granted by the University of Arizona Institutional Review Board (UA-IRB) as well as approvals obtained from each hospital, including the general directory of health affairs in Eastern Saudi Arabia. Each hospital had specific characteristics as follows.

#### 2.1.1. Public Hospital

This hospital is one of the main public hospitals in the region, with a capacity of 500 beds. The facility has several other outpatient clinics, such as dermatologic, internal medicine, and orthopedics, and committees established to address infection control and organ transplants.

#### 2.1.2. Security Forces Hospital

This hospital serves security force personnel and their families. It has 132 beds and some outpatient clinics and other departments created to manage infection and environmental controls.

#### 2.1.3. Private Clinic

This clinic serves people who have insurance or pay for services *out-of-pocket*. It is a large facility with many outpatient clinics such as internal medicine, maternity, and otorhinolaryngology (ENT), but no hospitalization. Also, at the time of this study, it operated a 24-h emergency room in addition to its treatment room.

### 2.2. Participant Recruitment

Nurses and medical doctors were recruited from the outpatient and emergency room departments of each of the three hospital types. At each setting, the outpatient clinic supervisor provided a list of all the physicians and nurses assigned to work during the requested observation periods. Then, the sample population was selected through meetings with each healthcare worker on duty at each of the designated hospitals. During a weeklong visit at each setting, the premise of the study was explained to all healthcare workers, and participant permission was obtained to observe hand hygiene practices and to administer the self-report questionnaires. All doctors and nurses on duty in the emergency room were asked to participate. At the Security Forces hospital, infection control personnel assisted in providing access to healthcare workers on duty. Ultimately, 87 participants (46 medical doctors, 21 from the public hospital, 9 from the private clinic, and 16 from the Security Forces; and 41 nurses, 14 from the public hospital, 9 from the private clinic, and 18 from the security forces hospital) were recruited. Of the 87 participants who completed the questionnaire, 83 participants were observed during exams with at least three patients and hand hygiene practices were recorded using the checklist. No data were collected regarding patient behaviors, characteristics, reason for hospital visit, or diagnosis. Hand hygiene practices of healthcare workers (i.e., physicians and nurses) were evaluated before, during, and after interactions with patients, devices, and/or surrounding surfaces in each of the three Saudi Arabian hospitals.

### 2.3. Data Collection

Two instruments were utilized for this study, an Observation Checklist and Self-report Questionnaire.

#### 2.3.1. Observation Checklist

Direct observations of healthcare workers’ hand hygiene practices were conducted during work hours using a questionnaire, which contained 16 items, and was developed by the primary researcher’s application of the following three sources: CDC standards [17], WHO’s Five Moments for Hand Hygiene in Health Care [18], and Boyce and Pittet’s (2002) guidelines [19]. Items were grouped into three patient-related time-dependent categories: (a) contact with a patient prior to entrance into an examination room (Before), (b) contact during the exam (During), and (c) contact with a patient upon exit from an examination room (After).

#### 2.3.2. Self-Report Questionnaire

For the self-assessments, the questionnaire was reworded with the same 16 items from the observation sheet, making the statements active with a five-point Likert scale (i.e., ‘Never’, ‘Rarely’, ‘Every once in a while’, ‘Sometimes’, and ‘Always’) to allow healthcare workers to report their perceptions about their own hand hygiene practices after each scheduled observation in this study.

### 2.4. Data Analysis

#### 2.4.1. Person Correlation

Pearson correlation was used to examine data by the questionnaire regarding healthcare workers’ beliefs about hand hygiene practices obtained and their actual observed behavior of hand hygiene practices. In order to minimize the likelihood of missing data, all healthcare workers were provided work time to complete the questionnaire after being informed of the importance of the research. Data lacking both self-assessment and observations were excluded when testing the primary hypothesis, as a result, only data from the healthcare workers who were actually observed by the researcher and who had questionnaires completed were analyzed.

#### 2.4.2. *t*-test

Two independent sample *t*-tests were used to examine differences between hand hygiene practices among healthcare workers, two hospital types, and gender. A multivariate analysis of covariance (MANCOVA) was used to further assess the relationship. When using MANCOVA, the healthcare worker (physician or nurse) was the independent variable. The practice of hand hygiene measured under three conditions (before, during, and after) was the dependent variable. Statistical analysis controlled for department (emergency rooms or outpatient department), hospital type (public, private, or security forces), and gender (male and female) of the care provider.

## 3. Results

Sixty-four (53/83) percent of the observed participants completed the questionnaire, four completed the questionnaire without being observed. The participants selected for the study are summarized in Table 1.

### 3.1. Descriptive Statistics

**Figure 1:**
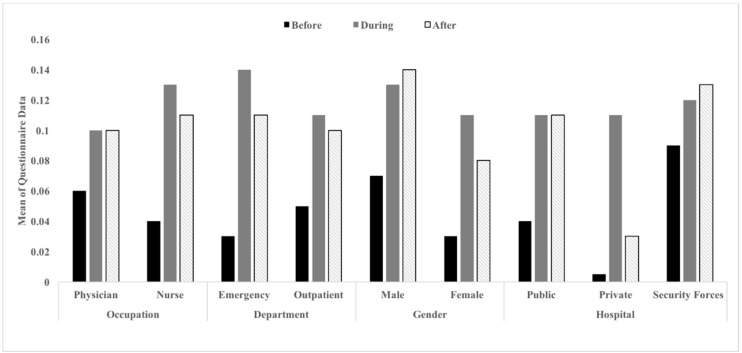
Comparing the mean scaled compliance scores of all variables based on the observation data. Note: Mean observation scores range from 0 to 1, with 1 being compliance 100% of the time.

**Figure 2:**
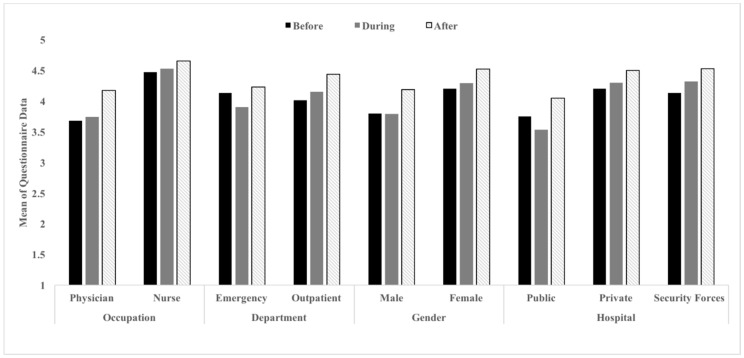
Comparing the means of all variables based on the questionnaire data. Note: Mean questionnaire scores range between 0 and 5, with 5 meaning compliance 100% of the time.

An overview of the data in descriptive statistics for selective variables is presented. Table 2 (observations) and Table 3 (self-reports) include minimum scores, maximum scores, means, and standard deviations of all variables, based on the observation and questionnaire data. Visual representations of the overall findings are shown in Figure 1 and Figure 2. The figures also indicate the percentage of observed hand hygiene compliance for physicians (27%) and nurses (29%) among the three time frames. In general, hand hygiene compliance scores before contacting patients among all variables were lower than compliance scores during and after contact with patients. Moreover, compliance scores during and after contact with patients were comparable to each other, except for the mean compliance score observed in the private clinic. In addition, the mean scores derived from the self-report questionnaire data were compared across conditions of patient contact. Based on the questionnaire data, all healthcare workers generally believed that they were compliant with hand hygiene procedures. Figure 1 and Figure 2 show that physicians appear to be more self-aware about their hand hygiene practices than nurses.

### 3.2. Person Correlation

The results of the Pearson correlation indicated that the healthcare workers’ beliefs of hygiene practices and the healthcare workers’ actual behaviors did not correlate, ranging from *r* = −0.01 to *r* = −0.20.

## 4. Discussion

The results of this study are robust and relevant to clinical practice in general and specifically in Saudi Arabia. First, the study design employed two observation methods (i.e., direct observation and self-report questionnaires). Second, conduct of this study increased the awareness of hand hygiene issues in healthcare settings in Saudi Arabia. Third, this study evaluated compliance with hospital hand hygiene policy in multiple types of Saudi Arabian hospitals in Eastern Saudi Arabia and considered the role played by healthcare settings and practice differences, since doctors tend to stay in a single office and patients rotate through each doctor’s office. 

The study had three main findings. First, hand hygiene compliance was considered low among healthcare workers in Saudi Arabia for physicians (27%) and nurses (29%), with no statistical difference observed between the two. This finding is consistent with the literature. According to Caglar et.al [14], an extensive literature review about hand hygiene practices among healthcare workers indicated that only 12.9% to 56% were compliant. For the self-report questionnaires, nurses reported higher hand hygiene compliance than physicians. Similarly, Harris et al. [20] found that non-physicians reported a higher compliance with hand hygiene when compared to physicians. Healthcare workers’ beliefs, based on self-report questionnaire responses, and actual behaviors, as noted by the primary researcher’s observations, did not correlate. This element of the data implied that the healthcare workers believed themselves to be more compliant with hand hygiene procedures than they actually were. A possible explanation for this discrepancy could be ignorance of hand hygiene protocols or failure to be aware of their own hand hygiene behavior.

When comparing the results from data collected in the public hospital to the data from the security forces hospital, no statistical differences existed. This is of note because the public hospital had a dedicated MERS hand hygiene intervention campaign, while the security forces hospital only had an annual seminar regarding hand hygiene procedures. This suggests that the campaign to increase hand hygiene in the public hospital needs to be rethought with a different implementation strategy. Antoniak’s findings supported this idea that changes in hand hygiene behavior were not sustained beyond the period of educational intervention, demonstrating that education is not significantly effective [21]. Results from the private clinic showed the lowest compliance rates, possibly due to absence of hygiene practice materials and resources, like alcohol-based hand sanitizers in the examination rooms.

In this study, a single person was assigned to observe the hand hygiene practices of each healthcare worker. While the design used in this study minimized inconsistencies among observations, it was difficult for just one observer to fully measure the proper protocol of hand hygiene (i.e., duration and technique). Future researchers could use either video recordings or two or more observers to collect a more accurate representation of the behaviors of each participant. The premise of the study was explained fully to the study participants prior to observation sessions. This prior knowledge could have added bias to the study results by increasing rates of compliance. However, the results indicated very low hand hygiene compliance rates. This could signify that either the participants actually performed their usual practices with patient exams or that though they tried to comply due to the observer in the room they were unsuccessful because they had not had proper training. Neither of these explanations would be conclusive unless future studies of the participants’ hand hygiene knowledge are conducted.

Scientific research has proven that hand hygiene is an important tool and the most effective way to prevent the spread of infectious diseases [11]. Unfortunately, prior to this study, hand hygiene practices in Saudi hospitals had not been investigated and no published articles were found. Throughout the study, the primary researcher observed many unexpected behaviors that infection control personnel at all hospitals should note and address. For example, in one observation, a healthcare worker washed her hands, then used a tissue to close the water handle, and then used the same tissue to clean nearby surfaces. On other observations, a few healthcare workers were observed not implementing proper alcohol application protocol, by using a tissue to dry excess alcohol on their hands. In other cases, some healthcare workers used alcohol wipes to clean their hands, not after hand-washing, as deemed in the proper protocol, clearly contributing to the lack of hand hygiene compliance. These behaviors should be further documented and referenced in order to improve the education of healthcare workers in the practice of proper hand hygiene.

## 5. Conclusions

In summary, no statistically significant correlations were found between healthcare workers’ reports of hand hygiene practices and the observed behaviors of hand hygiene practices. Additionally, there were no statistically significant differences among the two types of healthcare workers (i.e., nurses and physicians) when observed. However, there were statistically significant differences when comparing the self-reported questionnaire data to observations of physicians’ and nurses’ hand hygiene practices. These results demonstrate that healthcare workers likely understand the importance of hand hygiene, but in practice fail to implement appropriate hand hygiene in routine activities.

Future research should utilize surrogate viral tracers to appropriately track virus transmission and portability in healthcare environments in Saudi Arabia. This is crucial for public health researchers to fully understand exposure probabilities and infection risks as well as the role of hand hygiene in the reduction of infection in order to create more focused and effective interventions. Additionally, the significant differences between physicians’ and nurses’ self-reports regarding hand hygiene compliance suggest further targeted inquiry and experimental research is needed to fully explore these large discrepancies.

## Figures and Tables

**Table 1:** Participant Information.

Hospital	Observation			Questionnaire		
	Physicians	Nurses	Total	Physicians	Nurses	Total
**Public**	20	14	34	12	3	15
**Private**	8	9	17	5	5	10
**Security Forces**	16	16	32	12	16	28
**Total**	**44**	**39**	**83**	**29**	**24**	**53**

**Table 2:** Two Independent Sample *t*-Tests of the Observational Data on Hand-Hygiene Practices. (Observations)

Physicians and Nurses							
Hand Hygiene	Physicians Descriptive Statistics		Nurses Descriptive Statistics				
	M	SD	M	SD	*df*	*t*	*p*
**Before**	0.06	0.14	0.04	0.12	81	0.69	0.49
**During**	0.10	0.11	0.14	0.13	81	−1.32	0.19
**After**	0.10	0.16	0.11	0.15	81	−0.17	0.87
**Total**	0.27	0.27	0.29	0.26	81	−0.37	0.72

Note: M = Mean; SD = Standard Deviation; *df* = Degree of Freedom; *t* = *t*-statistic; *p* = probability value

**Table 3:** Two Independent Sample *t*-Tests of the Questionnaire Data on Hand-Hygiene Practices. (Self-reports)

Physicians and Nurses							
Hand Hygiene	Physicians Descriptive Statistics		Nurses Descriptive Statistics				
	M	SD	M	SD	*df*	*t*	*p*
**Before**	3.68	1.13	4.47	0.63	51	−3.04	0.004
**During**	3.74	0.94	4.53	0.48	51	−3.72	0.000
**After**	4.17	1.01	4.65	0.54	51	−2.10	0.041
**Total**	10.19	4.57	13.65	1.51	51	−3.56	0.001

Note: M = Mean; SD = Standard Deviation; *df* = Degree of Freedom; *t* = *t*-statistic; *p* = probability value.
